# Design and Preparation of New Multifunctional Hydrogels Based on Chitosan/Acrylic Polymers for Drug Delivery and Wound Dressing Applications

**DOI:** 10.3390/polym12071473

**Published:** 2020-06-30

**Authors:** Ioana A. Duceac, Liliana Verestiuc, Cristina D. Dimitriu, Vasilica Maier, Sergiu Coseri

**Affiliations:** 1 “Petru Poni” Institute of Macromolecular Chemistry of Romanian Academy, 41 A Gr. Ghica Voda Alley, 700487 Iasi, Romania; coseris@icmpp.ro; 2 Department of Biomedical Sciences, Faculty of Medical Bioengineering, “Grigore T. Popa” University of Medicine and Pharmacy, 9-13 M. Kogalniceanu Street, 700454 Iasi, Romania; 3 Department of Morpho-Functional Sciences, Faculty of Medicine, “Grigore T. Popa” University of Medicine and Pharmacy, 16 Universitatii Street, 700115 Iasi, Romania; daniela.dimitriu@umfiasi.ro; 4 Department of Textiles and Leather Chemical Engineering, “Gheorghe Asachi” Technical University of Iasi, 700050 Iasi, Romania; vmaier@tuiasi.ro

**Keywords:** superabsorbent hydrogel, *N*-citraconyl-chitosan, poly(acrylic acid)/poly(methacrylic acid)

## Abstract

The dynamic evolution of materials with medical applications, particularly for drug delivery and wound dressing applications, gives impetus to design new proposed materials, among which, hydrogels represent a promising, powerful tool. In this context, multifunctional hydrogels have been obtained from chemically modified chitosan and acrylic polymers as cross-linkers, followed by subsequent conjugation with arginine. The hydrogels were finely tuned considering the variation of the synthetic monomer and the preparation conditions. The advantage of using both natural and synthetic polymers allowed porous networks with superabsorbent behavior, associated with a non-Fickian swelling mechanism. The in vitro release profiles for ibuprofen and the corresponding kinetics were studied, and the results revealed a swelling-controlled release. The biodegradability studies in the presence of lysozyme, along with the hemostatic evaluation and the induced fibroblast and stem cell proliferation, have shown that the prepared hydrogels exhibit characteristics that make them suitable for local drug delivery and wound dressing.

## 1. Introduction

Hydrogels are a momentous collection of materials with highly diverse applications in engineering, biomedical and pharmaceutical sciences [1]. Among these, drug delivery and wound dressing are specifically of interest for scientists due to the increasing number of patients having various types of acute or chronic wounds (surgical, ulcers or burns that need emergency or constant, long-term medical assistance) combined with a considerable economic burden. The significance of this problem is easily illustrated by the growing wound dressing market share and size, from USD 7.1 bln. of the global market in 2019 to an estimated USD 12.5 bln. by 2022 [2].

An effective treatment is a constant challenge which leads to a careful selection of materials in medical practice and requests an imperative development of new advanced wound dressings with combined properties. These smart materials are able to absorb blood and wound fluids, protect the injury, accelerate the healing process by one or more mechanisms: promoting fibroblast proliferation and keratinocyte migration, which are both necessary for complete epithelialization; prevention of the wound contamination with opportunistic pathogen species; efficient transport of biologically active molecules (e.g., antimicrobial agents and other pharmaceuticals) [2].

Aiming at products that could be marketed, one of the research strategies focuses on multifunctional advanced dressings. These smart, high performance materials in the form of superabsorbent hydrogels, are expected to show various characteristics and fulfil several demands for application as wound dressing, such as to provide a porous structure required to absorb exudates, while maintaining a moist environment; a high swelling ratio and fluid retention, which entail the ability to rehydrate dry wounds (e.g., eschars); transparency to allow wound monitoring; offer the possibility to endow bioactive molecules in their matrix; good biodegradability, biocompatibility, and hemocompatibility [3,4].

Furthermore, the wound healing process is improved by using the same hydrogel system for the additional delivery of a therapeutic payload, such as antibiotics, anti-inflammatory drugs or growth factors [5]. Given the premises that hydrogels enable large amounts of bioactive molecules to be loaded into the polymeric network, they permit controlled release at the desired site as the hydrogels are placed directly at the targeted location (like a dressing or injectable/in situ gelling material), and the drug release occurs through a specific mechanism or strategy (swelling, network relaxation, temperature-induced transition, etc.), it can be hypothesized that hydrogels can be used as dressings able to perform controlled drug delivery [6].

The challenge of multifunctional hydrogel design and preparation consists of finding the optimal formulation that yields in the material with the best performance as both a drug delivery system and wound dressing. Such a macromolecular structure can be obtained by using a wise selection from the large variety of natural and synthetic polymers able to provide good biological interactions and modulated mechanical resistance and biochemical interactions [7].

Chitosan, a copolymer which consists of β-(1→4)-linked 2-acetamido-2-deoxy-D-glucopyranose and 2-amino-2-deoxy-D-glucopyranose units, is one of the most abundant polysaccharides in nature and possesses specific properties highly required for materials suitable in drug delivery, wound healing, and, ultimately, in regenerative medicine. This natural macromolecule is widely used for hydrogel fabrication due to its intrinsic properties such as excellent biocompatibility, low toxicity and immune-stimulatory activity [8], leading to positive effects on wound healing [9]. However, chitosan is poorly soluble in water and in the common organic solvents, except in aqueous acidic medium. To overcome this drawback, chitosan is often subjected to chemical modifications [10]. Chitosan is already the major component of various commercial wound dressings with hemostatic activity, such as ChitoSAM™ (SAM Medical, Wilsonville, OR, USA, 2018), ChitoGauze XR pro (North American Rescue, Greer, SC, USA, 2018), ChitoFlex (H.M.T. Inc., The Woodlands, TX, USA, 2007), and Axiostat® (AXIO, New York, NY, USA, 2018), which recommends this polymer to be further investigated for this type of application [11].

On the other hand, poly(acrylic acid) (PAA) and poly(methacrylic acid) (PAM) are synthetic polymers with biocompatible and antibacterial properties, and, therefore, are widely used as (bio)adhesives or superabsorbent materials [12,13,14]. Polymers grafted with acrylic acid yield in highly hydrophilic materials and tunable scaffolds for drug delivery systems due to the presence of pendant carboxylic groups [15,16,17,18]. In other words, acrylic acid-based hydrogels make excellent wound dressings due to their ability to retain large volumes of water and water-soluble drugs loaded into their matrix [19].

This study focused on the design, synthesis and advanced characterization of novel multifunctional hydrogels, having a specific composition optimized according to the targeted applications. Thus, a series of hydrogels based on *N*-citraconyl-chitosan and acrylates with various structures has been obtained, and their level of performance was comparatively assessed. The variation of two parameters was considered for the optimization: (i) the nature of the cross-linking agent, either PAA or PAM, and (ii) the initiator ratio used for free radical polymerization. In addition, L-arginine, an amino acid which is a key factor in accelerated wound healing and in cellular recognition and adhesion, was conjugated with the hydrogels network in order to modulate their interaction with various fluids and contact with cells. The influence of different components on the ability of hydrogels to respond to relevant biological media was also investigated. Moreover, the hydrogels capacity to act as a drug carrier and delivery system was evaluated by studying the release profiles and kinetics of ibuprofen, a nonsteroidal anti-inflammatory drug.

## 2. Materials and Methods 

### 2.1. Materials and Hydrogel Preparation

Chitosan (MW 80 kDa, Sigma-Aldrich, Darmstadt, Germany) was purified by the solubilization–precipitation method. Briefly, chitosan was solubilized in 5% aqueous acetic acid solution, and then, precipitated with 20% NaOH solution, centrifuged and dialyzed against distilled water. Citraconic anhydride, acrylic acid (AA), methacrylic acid (AM), ammonium persulfate (APS), *N,N,N′,N′-*tetramethylethylenediamine (TEMED), l-arginine, 1-ethyl-3-(3-dimethyl-aminopropyl)carbodiimide (EDC), *N*-hydroxysuccinimide (NHS) and ibuprofen (Ib) were purchased from Sigma-Aldrich, Germany. AA and AM were purified by passing through an inhibitor removal column (HQ, Sigma-Aldrich, Germany) and APS was recrystallized from methanol. For the cytotoxicity assays, the following were used: Hank’s Balanced Salt Solution (HBSS), Dulbecco’s Modified Eagle’s Medium (DMEM), thiazolyl blue tetrazolium bromide (MTT) and calcein AM (Sigma-Aldrich, Germany). All other solvents and chemicals were obtained from Sigma-Aldrich, Germany, and were used as received.

#### 2.1.1. Chitosan Chemical Modification

Chitosan was chemically modified with citraconic anhydride by the *N*-acylation reaction using a previously described method. [20] Briefly, 27.5 mL citraconic anhydride were solubilized in 40 mL acetone and the solution was added dropwise under stirring to a chitosan solution (1% w/wt. in 5% acetic acid aqueous solution) in the presence of 80 mL methanol, to prevent chitosan precipitation in the presence of acetone, and has been allowed to react at room temperature (25 °C), for 18 h. The chitosan/anhydride molar ratio was 1:15, chosen based on our previous studies [20]. The obtained product was further purified through dialysis against distilled water for 7 days and then freeze-dried.

#### 2.1.2. Hydrogels Preparation

The chemical method of hydrogel preparation is the copolymerization of citraconyl-chitosan with AA or AM by free radical polymerization with the formation of cross-linked networks. Thereby, a stock solution of 1% citraconyl-chitosan in distilled water was prepared and the necessary volume for each hydrogel was transferred. AA or AM, followed by APS and TEMED, were added to the chitosan solution under vigorous stirring at 800 rpm and allowed to homogenize for 10 min. In Table 1, the hydrogels composition is presented in terms of synthetic monomer/initiator ratio, which is the variable for the hydrogels. The AA and AM volumes are not given in the table, as it is a constant ratio between the natural and synthetic polymers of 1:7 *w/w* citraconyl-chitosan/synthetic monomer. The APS/TEMED thermolabile initiator system was chosen due to its low toxicity and it was added to the polymerization mixture in different ratios, as presented in Table 1. The mixtures were transferred into molds (10 mL glass cylinders) and the radical polymerization reaction was carried out for 2 h at 70 °C. The obtained hydrogels have been thoroughly washed in distilled water until constant pH (pH ≈ 6.5) to ensure the removal of all unreacted monomers, APS, TEMED, oligomers and other residues. The purified materials were freeze-dried for further characterization, thus, ensuring porous network conservation.

#### 2.1.3. l-Arginine Coupling with the Hydrogels Network

The amino acid coupling reaction evolved in two stages. Firstly, the weighted samples were immersed in specific volumes of EDC/NHS solution buffered at pH = 5.4 for 6 h, for the activation of carboxylic groups. The pH value was chosen to ensure the availability of COOH groups from the synthetic polymer chains for the coupling reaction. The EDC/NHS solution volumes were determined for each sample based on the molar ratios COOH:EDC = 1:2, EDC:NHS = 1:1, and on the concentration of COOH groups for each hydrogel calculated from the elemental analysis data. Then, all hydrogels were washed three times with PBS, and then, put in contact with the arginine solution, at pH = 10 for 24 h, to enable the formation of the amide bond between the activated carboxylic groups from hydrogels and the α-amino moieties in the structure of arginine. Finally, the functionalized scaffolds were washed thoroughly with PBS and distilled water, then freeze-dried.

### 2.2. Hydrogels Characterization

#### 2.2.1. Structural and Morphological Analysis

The chemical composition was evaluated by elemental analysis, quantifying the nitrogen content in hydrogels by the Kjeldahl method. All FTIR spectra were recorded on dried samples in KBr pellets using a Bruker Vertex 70 spectrophotometer (Berlin, Germany) and scanned within the range 400–4000 cm^−1^ in transmittance mode. Gold coated cross-sections of hydrogels were examined with a SEM Tescan-Vega microscope (Brno, Czech Republic), at ambient temperature, with an operating voltage of 30 kV, and the morphology images were analyzed with ImageJ software.

#### 2.2.2. Fluid Retention Study

The swelling behavior of all hydrogels was tested in phosphate-buffered solution (PBS) and simulated wound fluid (SWF), using the gravimetric method (50 mg aliquots immersed in 10 mL solution, incubated at 37 °C). The PBS solution had precise parameters: pH = 7.4, density 1.02 g/mL and concentration 0.01 M. The SWF solution was prepared using albumin (2%), NaCl (0.4 M), CaCl_2_ (0.02 M) and was finally buffered at pH = 7.4 with Trisbase (0.08 M); all components were solubilized in deionized water. Measurements were made for all aliquots at regular intervals and the swelling degree was calculated using the following equation:

(1) $$SD=\frac{W_{t}-W_{0}}{W_{0}}\times 100$$

where *SD*—swelling degree (%), *W_t_*—hydrogel weight at different times and *W*_0_—initial hydrogel weight.

Before each gravimetric measurement, the swollen aliquots were gently tapped on absorbing paper to eliminate the surface liquid excess. The tests were performed in triplicate.

#### 2.2.3. Drug Release Study

Ibuprofen was used as a model for drug release study. Initially, hydrogels were loaded by the swelling-diffusion method [21,22]. Weighted dry aliquots of 80 mg were immersed in 10 mL of 8 mg/mL ibuprofen solution in water/ethanol (1:1 *v/v*). After 24 h, the excess was removed and the samples were freeze-dried (using a freeze-dryer with pump for solvents, Labconco, USA). The in vitro release study was performed at 37 °C and neutral pH, chosen to mimic the extracellular environment. The aliquots were introduced in dialysis membrane and immersed in 500 mL PBS (pH = 7.2, 0.035 M), under dynamic conditions, and the samples were analyzed. The release was studied for 600 min and readings were performed automatically at preset time intervals. The absorbance was recorded at 220 nm, using an Erweka Dissolution tester DT 700 (Germany) coupled with a PharmaSpec UV-1700 spectrophotometer (Shimadzu, Japan). Blank experiments using drug-free hydrogels confirmed that there was no interfering background absorption. The released amount of ibuprofen was calculated using a standard calibration curve measured for the drug at 220 nm on a dilution series. Finally, the cumulative amount of released drug was plotted against time. Data analysis was done by fitting various kinetic models to the curves.

#### 2.2.4. In Vitro Degradation Study

Aliquots of the materials (30 mg) were immersed in 10 mL PBS, pH = 7.4, 0.01 M, containing 1200 μg/mL 1ysozyme, in a dialysis membrane (MWCO = 14,000 Da) that was further immersed in 30 mL PBS and incubated at 37 °C. For measurements, 1 mL of solution was sampled and evaluated. The concentration in saccharide reducing units was measured by the potassium ferricyanide method [23]: the extracted solution was mixed with 4 mL of 0.05% potassium ferricyanide solution in 0.5M Na_2_CO_3_ solution, and boiled for 15 min, allowed to cool and then, diluted with 5 mL PBS. The sample absorbance was measured at 420 nm, employing a PharmaSpec UV-1700 spectrophotometer (Shimadzu, Japan) and the amount of chitosan reducing ends was calculated using a calibration curve for *N*-acetyl-d-glucosamine.

#### 2.2.5. In Vitro Cytocompatibility Study

Selected hydrogels (synthesized with 0.8%, 1.2%, and 1.4% APS) were sterilized by immersion in 5 mL of 70% ethanol for 15 min and then, washed several times with HBSS and DMEM. The circular samples with 5 mm diameter cut with a sterile biopsy circular scalpel were incubated with normal human dermal fibroblasts (NHDF, Lonza, Basel, Switzerland), in 48-well plates and, as a reference, cells were seeded under the same conditions. Cell viability was assessed at 24, 48 and 72 h using a direct contact MTT assay. The absorbance of the obtained solutions was measured at 570 nm using a Tescan Sunrise Plate Reader. Cell viability was calculated by Equation (2), where RMA is the relative metabolic activity, As is absorbance of the sample and Ac is absorbance of the negative control. Each result represents the mean viability ± standard deviation of four independent experiments.

(2) $$RMA=\frac{A_{s}}{A_{C}}\times 100$$

In addition to the MTT test, a live/dead staining assay was performed. Sterile hydrogel samples were put in direct contact with stem cells. At pre-set time intervals, the calcein solution was used to color the live cells by incubation for 20 min at 37 °C, followed by a microscopy analysis. Images were taken in phase contrast and fluorescence using a Leica DM IL LED Inverted Microscope with a Phase Contrast System and Fluorescence (Leica Microsystems GmbH, Wetzlar, Germany).

#### 2.2.6. Hemostatic Properties

The prothrombin time (PT) of blood and fibrinogen concentration in blood after contact with the prepared hydrogels were measured. Integral blood from healthy, non-smoker volunteers (venous puncture) was incubated with anticoagulant (aqueous sodium citrate 3.8% *w/v*; ratio 1/9 *v/v*). The hydrogels (5 × 10 × 1 mm) were added to 5 mL blood, and the control sample was considered the free integral blood, and both the sample and control were incubated at room temperature for 30 min. After that, the hydrogels were separated from blood by centrifugation (2500 rpm, 10 min). PT in blood plasma was determined (as a mean of three values) using a semi-automatic coagulometer Helena with 2 channels and photo-optical technique coagulation systems and PT-Fibrinogen kit (International Sensitivity Index (ISI) = 1.07). The International Normalized Ratio (INR) was calculated as the ratio of prothrombin times recorded for the hydrogels (PTH) and control (PTC) samples, raised to the power of the ISI value:

(3) $$INR={\left(\frac{PTH}{PTC}\right)}^{ISI}$$

## 3. Results and Discussions

### 3.1. Hydrogels Structure

**Figure 1:**
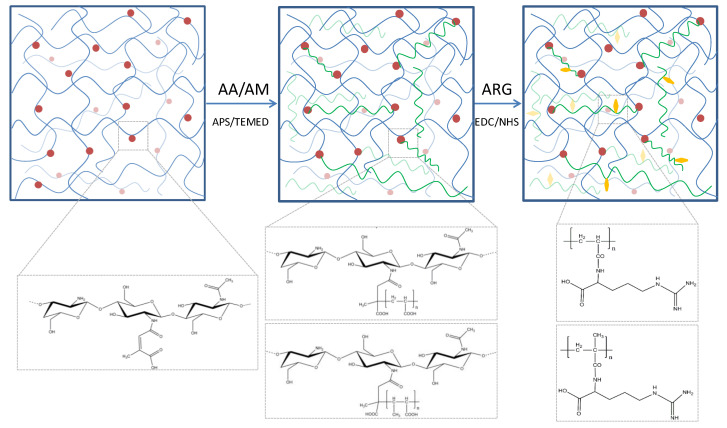
Schematic representation of the hydrogel network.

The design of chitosan-based hydrogels considered the parameters that influence the formation of the cross-linked polymeric networks. A schematic illustration of the hydrogel preparation is illustrated in Figure 1. In the first stage, chitosan was chemically modified with citraconic anhydride. The anhydride reacted with the amino groups available on the chitosan backbone and yielded in amide bonds, which induced novel useful properties. The C=C bonds from the anhydride moieties are available for further copolymerization.

In order to observe and compare the hydrogels structure and properties depending on the acrylate nature, a constant 1:7 ratio (*w/w*) between citraconyl-chitosan and the synthetic monomer was chosen for both AA- and AM-containing materials.

The free radical polymerization in the second stage was initiated by the thermal decomposition of APS molecules, in the presence of TEMED, at 70 °C, when the free radicals reacted with either monomer molecules, AA or AM, or with citraconyl sequences in the chitosan structure. Consequently, it can be assumed that the network was finally formed of (1) PAA or PAM cross-links between *N*-citraconyl-chitosan chains, as designed, (2) *N*-citraconyl-chitosan/PAA or PAM graft copolymer, and (3) a semi-IPN of citraconyl-chitosan and PAA or PAM. All polymeric chains have been stabilized through hydrogen bonds, ionic interactions between –COOH and –NH_2_ groups, or other physical interactions.

From the material science point of view, the aim of this study was to assess the influence of APS concentration on the hydrogel cross-linking process. The APS percentage is reflected in the cross-linking density of the polymeric network, due to the occurrence of more or less reaction centers. The acrylic chain length further affects the hydrogel morphology, pore dimension, fluid absorption and retention etc. The cross-linking reaction yield was between 58 and 79% for hydrogel with PAA and in the range 41–59% for those with PAM (data in Table 1). A correlation can be observed between the polymerization yield and the initiator ratio: the yield decreased along with the increase in APS concentration in the reaction mixture. Moreover, the values were lower for PAM polymerization, but the value range was similar for both synthetic polymers.

Finally, in order to achieve improved biological interactions, the hydrogels were optimized by the bioconjugation of l-arginine, an amino acid that could be coupled with the carboxylic groups existing on the synthetic polymer chains in both PAA and PAM. l-arginine is an amino acid precursor of proline which is converted to hydroxyproline and then, to collagen; it has a positive influence on the insulin-like growth factor (IGF-1), a hormone which promotes wound healing. Given its properties, l-arginine is expected to impart valuable characteristics to the conjugated materials. Consequently, the properties were assessed prior and after this reaction in order to compare the hydrogels performance and arginine influence.

**Figure 2:**
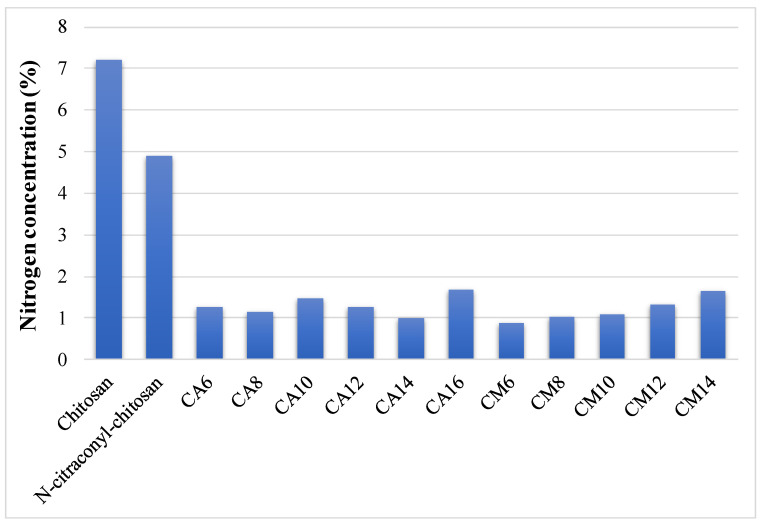
Nitrogen concentration of chitosan, *N*-citraconyl-chitosan and prepared hydrogels.

The elemental analysis was performed on chitosan, citraconyl-chitosan and all hydrogel samples, before the arginine conjugation reaction. The composition was calculated and the data are shown in Figure 2.

Compared to raw chitosan, the modified polysaccharide contained less nitrogen, thus, confirming the presence of citraconic anhydride in its structure. The decrease in nitrogen percentage is more significant in hydrogel samples due to the acrylic cross-links. The amount of nitrogen increased along with APS ratio in the hydrogels prepared with AM, indicating a higher reaction yield when less initiator was used. These data allowed the determination of the final composition in each hydrogel and the content of chitosan and synthetic polymer in 100% hydrogel is presented in Table 1. Based on these results, the carboxylic groups available for the final arginine coupling reaction could be determined.

**Figure 3:**
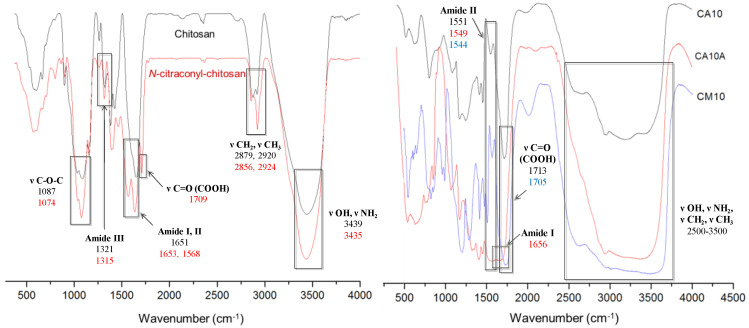
FTIR spectra for chitosan, *N*-citraconyl-chitosan (left), and selected hydrogels (right).

The comparative FTIR spectra recorded for chitosan and citraconyl-chitosan, and for the selected scaffolds CA10, CA10A and CM10, respectively, are presented in Figure 3.

Chitosan exhibits specific absorption bands in the FTIR spectrum, as follows: one intense absorption peak at 3439 cm^−1^ assigned to the OH and NH_2_ functional groups present in the polysaccharides structure; the amide I band was present at 1651 cm^−1^, shifted at higher wavenumber and combined with OH. The specific peak for C-O-C glycoside bond appeared at 1087 cm^−1^.

By comparison, the spectra of citraconyl-chitosan displayed new peaks correlated to the new amide bonds and to the structure of citraconyl residue. Hence, a large intense band was recorded between 1709 and 1568 cm^−1^ as a result of the fusion of the peaks specific to amide I and the carboxylic group, due to C=O stretching. Furthermore, C-O-H in plane bending from the COOH group led to the peak at 1461 cm^−1^, while the peaks at 2924 and 2856 cm^−1^ were correlated to the methyl groups symmetric and asymmetric stretching vibrations.

Scaffolds with 1% APS, cross-linked with AA or AM, before and after arginine conjugation, are presented in Figure 3 (right). The chemical cross-linking was confirmed by the presence of intense peaks at 1713 cm^−1^ (CA10) and 1705 cm^−1^ (CM10), correlated with the C=O bond from the carboxylic group and the intense frequency bands in the interval 2500–3500 cm^−1^ specific to PAA–O–H bond stretching vibrations (from the carboxylic group); in the range 2600–3600 cm^−1^, for the hydrogels with PAM, the bands were more intense due to, and shifted by, the symmetric and asymmetric stretch specific to the methyl group. In addition, the presence of the amide bond previously formed between chitosan and the anhydride was confirmed by the signals at 1551 cm^−1^ (CA10) and 1544 cm^−1^ (CM10), bands assigned to N–H bending vibrations and C–N stretching specific to amide II, hence, confirming polymer modification.

Arginine coupling with hydrogels yielded in new peaks that appeared in the FTIR spectrum of sample CA10A, corresponding to the amide bond vibrations, as follows: at 1656 cm^−1^ a new peak related to the stretching vibrations of C=O bond (amide I); at 1549 and 1323 cm^−1^, respectively, novel bands assigned to C–N stretching and N–H bending vibrations (amide II, III).

### 3.2. Hydrogels Morphology

**Figure 4:**
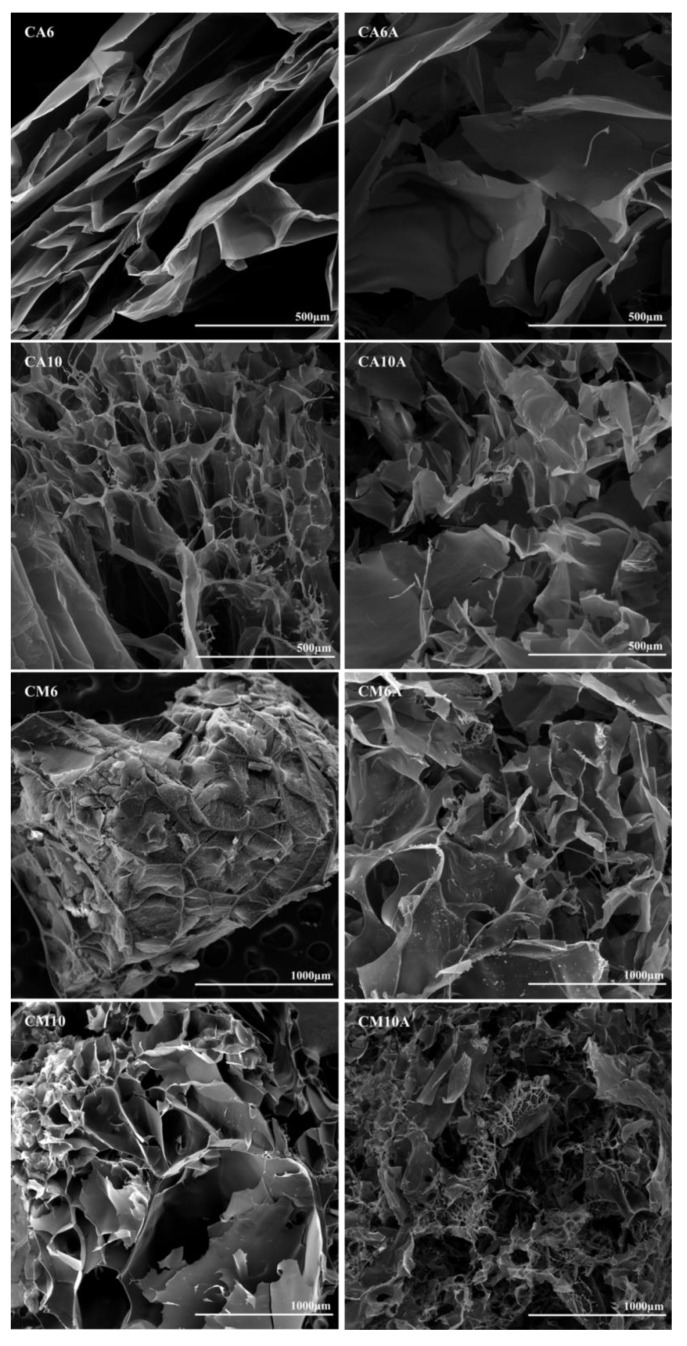
SEM images of hydrogels with acrylic and methacrylic acid, obtained with 0.6% or 1% APS, before and after arginine immobilization.

SEM images from Figure 4 were recorded for samples with AA and AM, synthesized in the presence of 0.6% or 1% APS, before and after arginine coupling, in order to compare and determine the influence of the considered preparation parameters. All samples displayed a porous morphology with interconnected pores of variable dimensions. At a microscopic level, there are areas that differ in aspect, which may be explained by the fact that the radical polymerization did not occur uniformly in the whole reaction volume, thus, leading to regions where more synthetic polymer was formed.

Particularly, for the AA-based hydrogels, well defined pores were obtained and the arginine coupling caused the formation of larger pores and high pore dispersity (see Table 2). However, in the case of hydrogels prepared with AM, the pore size increased with the increasing amount of APS and the presence of the amino acid favored the formation of pores with an even higher size (the average pore dimension varied between 40 and 550 μm). In the case of the arginine-containing hydrogels (sample CM10A in Figure 4), a significant decrease in pore size was noticed. The explanation resides in the direct bonding of arginine to the synthetic polymer moieties and, thus, arginine molecules are pendant toward the interior of the pores, which finally entailed the reduction in pore diameter.

### 3.3. Swelling Behavior in Simulated Physiological Conditions

**Figure 5:**
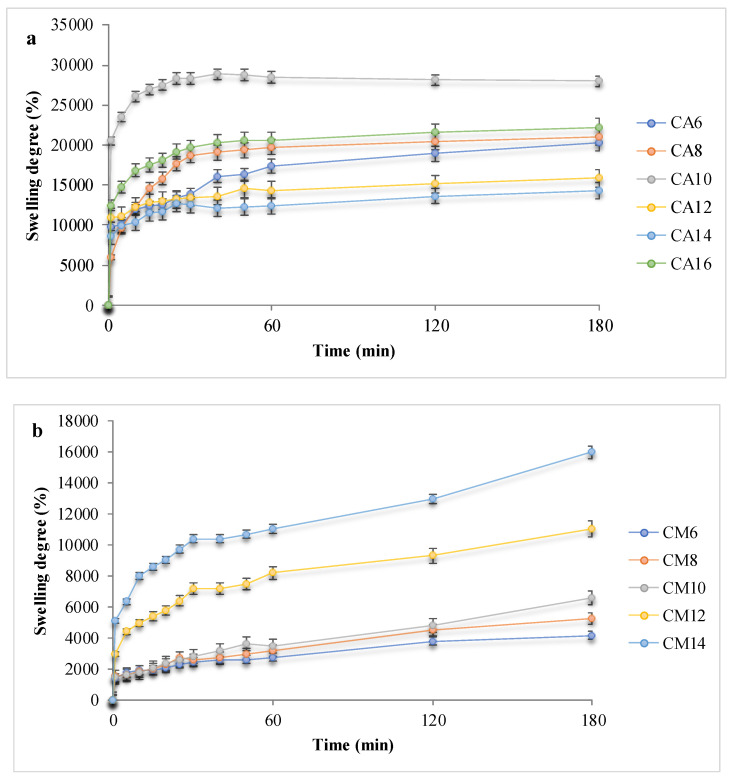

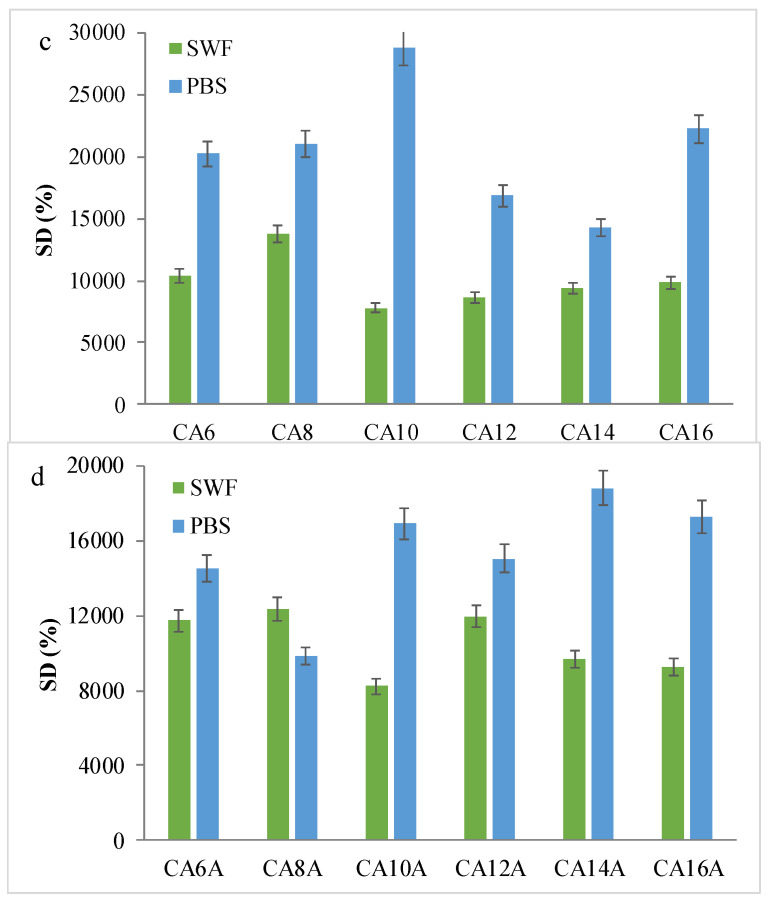
Swelling behavior of the hydrogels: (**a**,**b**) Kinetic swelling degree data, in PBS for hydrogels with acrylic and methacrylic acid; (**c**,**d**) Maximum swelling degrees in PBS or SWF, before and after arginine coupling.

The hydrogels’ ability to absorb fluids is essential in skin treatment applications, as it concerns, for example, the wound exudates or a hemorrhage. The swelling behavior of the hydrogels can be affected by multiple factors: the hydrophilic or hydrophobic functional groups (–COOH, –OH, –CO–NH–) existing on the polymer chains, the internal morphology, the network parameters and its elasticity, temperature, pH and swelling medium [7]. The experimental data obtained during swelling assays are shown in Figure 5. In order to test the hydrogels, two types of fluids were chosen according to various methods described in the literature: phosphate-buffered solution (PBS) at pH 7.4 and simulated extracellular or wound fluid (SWF) [24].

Hydrogels with AA showed SDs of up to 30,000% due to the hydrophilicity of the synthetic polymer and the maximum swelling degree, associated to the plateau, was reached 20–60 min after contact, depending on the polymer chain length and network morphology, but mainly due to the carboxylic groups that provide an important hydrophilic behavior. By comparison, the hydrogels with AM hydrated slower and absorbed up to 16,000%. APS influence can also be noted: a higher initiator concentration is associated with a higher swelling capacity and a slower absorption rate. Such a result is considered to be determined by a higher cross-linking density and shorter polymeric chains, which led to pores with smaller size. Hence, the network’s elasticity was altered, and the hydrogels became less flexible. [7] The presence of arginine in the polymeric networks determined a fast fluid absorption evident in all hydrogel samples, but with a smaller retained volume, up to 19,000% PBS. The influence of the swelling media was also investigated. The obtained experimental data suggested that SWF is absorbed in lower quantities as compared to PBS, presumably due to the high viscosity of the wound fluid and to existing albumin large molecules which do not access the domains with small pores, thus, leading to swelling degrees of a maximum of 12,000%.

Upon contact with any of the fluids, the polymer network started to swell progressively and the solution accessed the pores. The swelling mechanism may be either Fickian and the swelling is diffusion-controlled, or non-Fickian, when the swelling kinetics are based mostly on network relaxation, according to Ritger–Peppas model (Equation (4), data in Table 3) [25,26,27,28].

(4) $$\frac{M_{t}}{M_{\infty }}=kt^{n}$$

where *M*_t_ is the material weight at time *t*, *M*_∞_ is the initial material weight, *k* is the process rate constant, *t* is time and n is the diffusional exponent. The model was fit to experimental data for all hydrogels, before arginine conjugation, and tested in PBS.

In the present case, the diffusional exponent n had values between 0.75 and 0.93 for hydrogels with AA and between 0.61 and 0.73 for those with AM; hence, for all hydrogel samples the value of the diffusional exponent n fell within the range 0.45–1, a fact that indicates a non-Fickian swelling mechanism and substantiated that the water retention was driven by the relaxation of the network.

The fast swelling, favored by the presence of arginine, and the large volumes retained in the hydrogel networks, due to the chitosan/acrylate cross-linking, indicated a superabsorbent behavior [12,29,30,31,32,33,34] for the prepared hydrogels, which is a highly advantageous characteristic for applications such as drug delivery and wound dressing, as intended.

### 3.4. Ibuprofen Release Profiles and Kinetics

Ibuprofen (Ib), a nonsteroidal anti-inflammatory drug, is used for the treatment of pain, fever, and inflammation. It is insoluble in water, but has a high solubility in most organic solvents, including ethanol. The mechanism that leads to the analgesic, antipyretic, and anti-inflammatory effects evolves through a non-selective inhibition of cyclooxygenase (COX) enzymes. Normally, COX enzymes convert arachidonic acid to a prostaglandin, which further mediates pain, inflammation, and fever [35]. It is well known that Ib systemic administration causes severe issues, such as stomach and intestine injuries, circulatory problems (heart failure, AVC), and kidney lesions. To limit these drawbacks, a local delivery of Ib is preferred, such as a dressing. Moreover, in this case, the therapeutic effects are enhanced.

**Figure 6:**
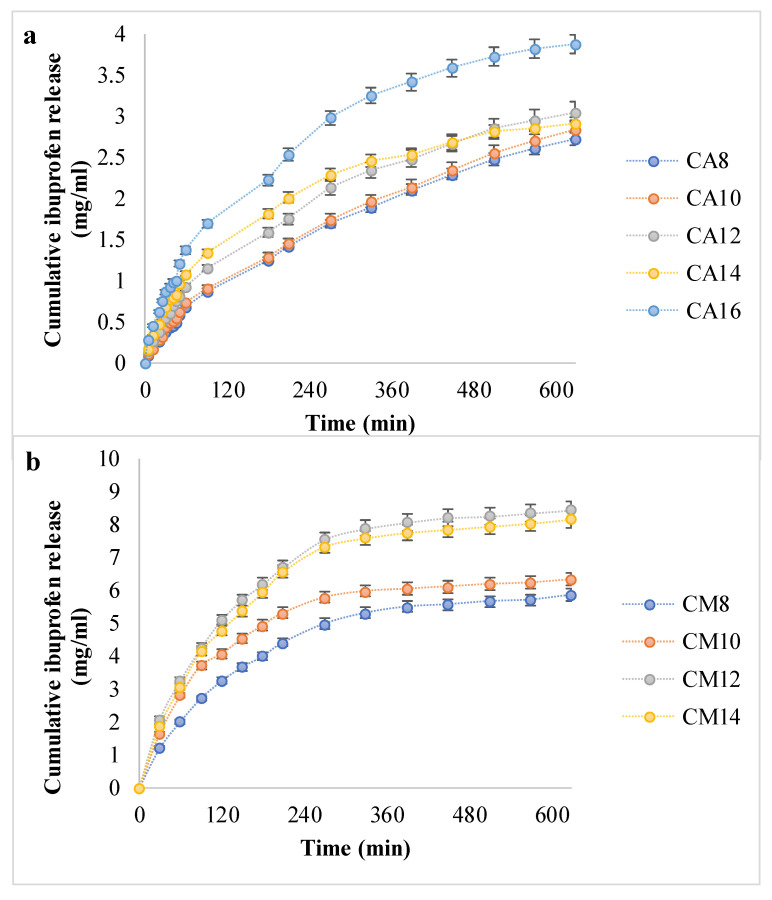
The in vitro ibuprofen release kinetic data (**a**,**b**).

The in vitro release of Ib from the hydrogels was analyzed in PBS, pH = 7.2, at 37 °C, mimicking the physiological environment. It is important to emphasize that the pH was maintained at the level recorded for the extracellular environment in physiological conditions, in order to be fit for the medical applications taken into consideration in the present article. The readings expressed as cumulative release were plotted versus time in order to obtain the drug release profiles and the results are shown in Figure 6.

The release profiles for the hydrogels with AA indicated a steady, controlled release of a maximum of 4 mg ibuprofen over 10 h. However, in the case of hydrogels with AM, Ib was released in double the amount and in a shorter time, similar to a burst effect. In addition, AA-based hydrogels did not reach a plateau after 600 min, while hydrogels with AM reached the plateau after 300–360 min, meaning that the equilibrium in drug concentration was reached between the hydrogel and the environment. Moreover, as the APS ratio grows from 0.8% to 1.4 or 1.6%, respectively, the amount of released Ib is greater. This can be explained by the fact that more APS led to larger pores, according to SEM micrographs, which favored drug diffusion in both directions—at loading and at release.

The drug loading and release capacity depend on the polymeric matrices’ structure and morphology, their ability to absorb and retain the drug solution, and the drug–hydrogel interaction [36]. The Ib molecule is amphiphilic and able to participate in various molecular/supramolecular associations. Ionic interactions between the COOH group from Ib and the NH_2_ moiety from chitosan were generally present in all hydrogels, since the same amount of chitosan was present in all matrices. It should be emphasized that there was no arginine in the hydrogels tested for drug delivery. The significant difference in hydrogel–drug interactions was correlated with the type of synthetic polymer.

Interestingly, the hydrogels with PAM released higher amounts of drug and in shorter time intervals, results that were in contradiction with the swelling degrees, since the PAA favored a faster absorption and the retention of larger fluid volumes. It is worth mentioning that the drug loading took place in a water/ethanol solution, which consequently may have been favorable for the swelling in chitosan/PAM networks, rather than in matrices with PAA. Moreover, it can be assumed that hydrophobic interactions appeared between Ib and the methyl groups in PAM structure.

It is known that the drug release from hydrogels is governed by several phenomena: diffusion, erosion, network relaxation, all in various proportions, depending on the polymer nature, network stability and parameters, morphology, hydrophilicity, the nature of the drug and its interaction with the matrix, the release medium etc., [37,38,39,40]. In order to study the drug release kinetics and mechanism, the drug release data were fitted into four models, using the following equations [21,41,42,43]:

(5) $$\mathrm{Zero}\;\mathrm{order}:\;\frac{Q_{t}}{Q_{0}}=k_{0}t$$

(6) $$\mathrm{First}\;\mathrm{order}:\;\ln\frac{Q_{t}}{Q_{0}}=k_{1}t$$

(7) Higuchi model: QQ0=kHt12

(8) $$\mathrm{Korsmeyer}–\mathrm{Peppas}\;\mathrm{model}:\;\frac{Q_{t}}{Q_{0}}=kt^{n}$$

where *Q_t_* is the amount of drug released at time t, *Q*_0_ is the original drug concentration in the material (40 mg), n is the release exponent and K is the release rate constant.

The models were fitted to the curves with the highest cumulative drug release in each series, namely CA16 (which had the highest APS ratio in the PAA-based hydrogels series) and CM12. The latter had a cumulative amount close to but higher than CA14, which had the highest APS ratio of the materials cross-linked with PAM. The results obtained for the correlation coefficient are shown in Table 4 and suggest that for the hydrogels with PAA, the release mechanism followed the Korsmeyer–Peppas kinetics [41], while the hydrogels with PAM fitted the Korsmeyer–Peppas and the Higuchi models well [42,43]. In addition to these data, it was important to determine the diffusional exponent n, which is a parameter that indicates the mechanism of drug release and varies depending on the geometry of the release device. Fickian diffusion is confirmed for *n* < 0.45; when 0.45 < *n* < 0.89, the drug transport is anomalous (non-Fickian)—in other words, the diffusion and relaxation rates are similar and the physical phenomena are diffusion and erosion of the matrix; if *n* > 0.89, then the key mechanism of drug release is Case II transport, thus, the drug release is determined by polymer relaxation [26,44]. The values obtained for the exponent n suggested anomalous (non-Fickian) transport, which indicated that the drug is released from the hydrogels by diffusion associated with the materials erosion. The drug release can be controlled by selecting the acrylate nature for the cross-linking and the initiator ratio. The drug is incorporated in a hydrophilic, initially glassy material, and the release is basically swelling-controlled [26].

### 3.5. The In Vitro Hydrogels Degradation

**Figure 7:**
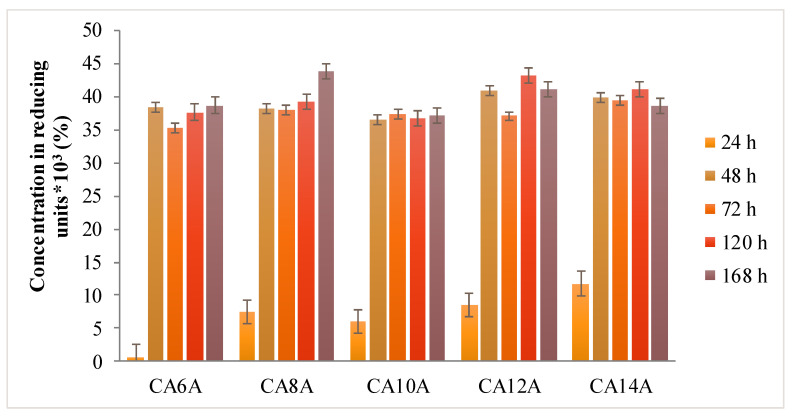
Degradation behavior of hydrogels in medium with enzyme.

The biodegradation assays have proven that the chitosan-based scaffolds were susceptible to lysozyme attack under simulated physiological conditions of pH and temperature, as shown in Figure 7.

The presence of the arginine molecules in the polymeric network caused the rapid absorption of the enzyme solution into the hydrogel networks, followed by their expansion, which entailed a fast degradation rate in all investigated samples, similar to a burst effect after 24–48 h. This phenomenon can be explained by the enhanced accessibility of lysozyme to the glycoside bonds within chitosan chains.

Lysozyme is present in any type of wound due to neutrophils secretion, in both infected and non-infected wounds. This phenomenon is associated with the inflammation phase of wound evolution and has the crucial role of cleaning the wound bed before any regeneration processes are triggered. The enzyme levels determined in the wound fluids were of 0.4 mg/mL for a wound with inflammation and at least 0.5 mg/mL lysozyme in an ulcer wound fluid. [45] Compared to the literature data, the 1.2 mg/mL lysozyme concentration used in this experiment was indicating that the hydrogels had a good stability in a more aggressive simulated environment, being able to maintain the cross-linked network for up to 48 h.

### 3.6. Hydrogels Cytocompatibility

**Figure 8:**
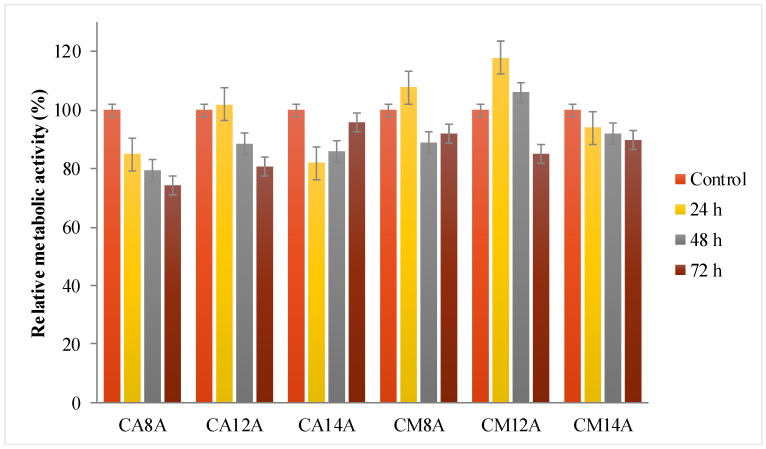
Relative metabolic activity data from the MTT assays for hydrogels with AA or AM, obtained with 0.8%, 1.2% and 1.4% APS, respectively, tested for different time intervals.

**Figure 9:**
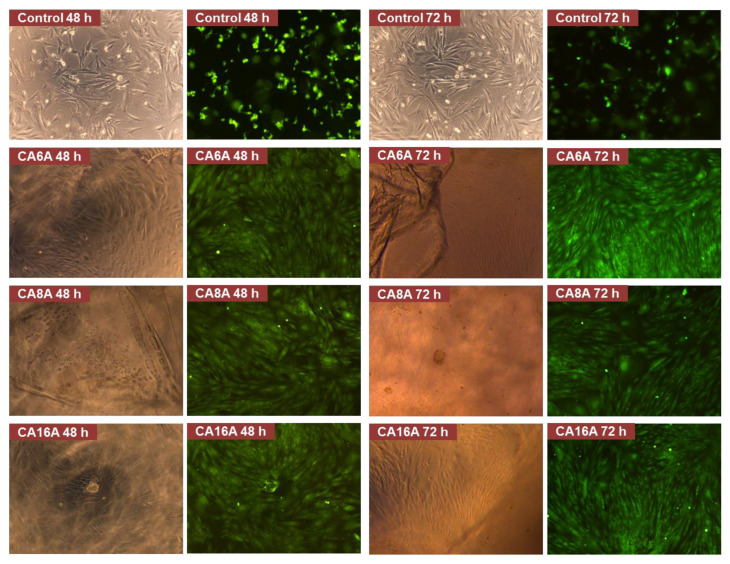
Live/dead staining assay images of negative control and cells after incubation with different hydrogels (magnification 10×).

All materials intended to be used for medical applications must be tested by means of biocompatibility. One of the standard assays is the MTT study for in vitro evaluation of cytocompatibility by the direct contact method. In addition to the obtained MTT data, a live/dead staining was performed and the results are shown in Figure 8 and Figure 9.

The relative metabolic activity induced by the hydrogels was between 76 and 100%, depending on the materials’ composition. All tested materials were conjugated with arginine, an amino acid well-known for its key role in accelerated wound healing and in cellular recognition and adhesion. Therefore, arginine is a valuable element in multicomponent systems used for drug delivery and wound dressing. [46] After the first 24 h, the best results were for materials with 1.2% APS, followed by a decrease in time, while the hydrogels with 1.4% APS showed a better cell response after a lag period of 72 h.

The fluorescence microscopy images were analyzed and several observations can be made: all hydrogels are transparent in the hydrated state, which can be an advantage for wound monitoring; the hydrogels induced an intense proliferation compared to the control with the formation of a confluent monolayer after 48 h; based on cell morphology, the stem cells appeared to have undergone differentiation. The cell response is the result of various cell–hydrogel interactions determined by the materials’ structure and properties on the one hand, and, on the other hand, by cell biology and biochemistry. Thus, it is possible that the inclusion of L-arginine in the matrices induced this cell behavior—the intense proliferation and the differentiation. Due to the fact that the differentiation is associated with a lower metabolic effect and that it may have begun after a fast proliferation—a process that lasted less than 24 h—it may explain the low RMA values.

### 3.7. Hemostatic Properties

The thrombogenic character is an important property for wound dressing materials. The protein adhesion initiates the coagulation cascade, and thus, the material can accelerate the thrombus formation, stop the hemorrhage, and stimulate the healing process. The hydrogels porosity and their rough surface are known to be beneficial to blood coagulation [47]. Moreover, the colloid formed on the pore’s surface upon the blood fluid sorption favors the adhesion of blood cells, and the dressing material presses the wound area and efficiently limits the bleeding as a physical barrier. The prothrombin time (PT) test is an efficient method to evidence the thrombotic or antithrombotic activity of biomaterials. Generally, the lower the value of PT, the faster the clotting rate is and the better the antithrombotic activity of these materials. [48] The experimental data recorded for the selected samples in terms of hemostatic activity are given in Table 5.

The excellent hemostatic properties of the prepared hydrogels were due to their strong swelling capacity and porous structure. For all tested hydrogels, the PT and INR values decreased in comparison with the control sample, while higher values for fibrinogen concentration were recorded. The PAA-based hydrogels exhibited the most intense hemostatic activity, compared with those with PAM, and this cumulative effect can be attributed to the synergic action of their specific characteristics: porosity, high hydrophilicity, and the positively charged moieties from chitosan and arginine immobilized onto networks, as was separately reported for chitosan-based materials and arginine [49,50,51,52,53,54]. Furthermore, at a lower APS ratio, the hemostatic effect was more intense for both hydrogel series. As superabsorbent hydrogels, these materials absorbed the plasma in the blood and, hence, promoted the local accumulation of the coagulation factors, erythrocytes and platelets, and accelerate the adhesion of the blood components to their surface, and thus, the blood coagulation was sped up. Therefore, taking into consideration these results and the literature data in the field, it can be stated that these new hydrogels successfully respond to the performance requirements for wound dressing materials.

## 4. Conclusions

In the present study, two series of hydrogels based on chemically modified chitosan grafted with acrylic polymers as cross-linkers were compared. The advantages of both natural and synthetic polymers led to hydrogels with combined desirable characteristics, and the networks were further conjugated with arginine in order to achieve enhanced properties. The results obtained from FTIR spectra, elemental analysis and SEM images confirmed the formation of networks with interconnected pores and evidenced a great influence on the hydrogel properties of the monomer and of the initiator ratio used during the synthesis. It has been confirmed that hydrogels have a high fluid absorption behavior after their interaction with buffer solution and simulated wound fluid, under physiological conditions. The ibuprofen release profiles were studied in vitro and the kinetics fitted the Korsmeyer–Peppas model best. The hydrogels have proved to be biodegradable in the presence of lysozyme with hemostatic properties and the cytocompatibility tests indicated the hydrogels’ ability to induce cell proliferation and differentiation. In conclusion, these superabsorbent hydrogels with tunable properties may be considered suitable materials for both drug delivery and wound dressing applications.

## Figures and Tables

**Table 1:** Hydrogels composition, the cross-linking reaction yield, and final chitosan and synthetic polymer content present in 100% hydrogel, as determined by elemental analysis.

Code *	Cross-Linker	Molar Ratio (%) AA/AM: APS/TEMED	Cross-Linking Reaction Yield (%)	*N*-Citraconyl-Chitosan (%)	Synthetic Polymer (%)
CA6	PAA	0.6	79.13	25.89	74.11
CA8		0.8	76.26	23.09	76.91
CA10		1	59.12	29.74	70.26
CA12		1.2	63.96	25.95	74.05
CA14		1.4	60.23	20.30	79.70
CA16		1.6	58.5	34.42	65.58
CM6	PAM	0.6	59.21	17.99	82.01
CM8		0.8	51.44	20.88	79.12
CM10		1	48.03	22.16	77.84
CM12		1.2	44.17	26.88	73.12
CM14		1.4	41.36	33.56	66.44

* an additional A was added to hydrogels code after the reaction with arginine.

**Table 2:** Pore size variations with hydrogel composition.

Hydrogel	Monomer Type	APS (%)	Presence of Arginine	Pore Dimension (μm)		
				Min	Average	Max
**CA6**	AA	0.6%	**✗**	55.2	148.0	448.4
**CA6A**		0.6%	**✓**	145.3	318.6	526.0
**CA10**		1%	**✗**	91.4	185.9	387.5
**CA10A**		1%	**✓**	150.4	240.1	372.6
**CM6**	AM	0.6%	**✗**	149.7	383.9	907.8
**CM6A**		0.6%	**✓**	287.6	539.8	899.4
**CM10**		1%	**✗**	251.0	548.5	1885.0
**CM10A**		1%	**✓**	13.6	41.8	73.1

**Table 3:** Kinetic parameters for hydrogels without arginine, immersed in PBS.

Hydrogel	*k*	*n*	Hydrogel	*k*	*n*
**CA6**	0.028	0.790096	**CM6**	0.0338	0.720515
**CA8**	0.0397	0.759804	**CM8**	0.0409	0.665587
**CA10**	0.0093	0.938351	**CM10**	0.0478	0.614805
**CA12**	0.0132	0.893475	**CM12**	0.0375	0.715687
**CA14**	0.0153	0.879649	**CM14**	0.0339	0.732957
**CA16**	0.0187	0.868381			

**Table 4:** Drug release correlation coefficient values from different kinetic models.

	Correlation Coefficient (r^2^)				Release Rate Constant, *k*	Release Exponent, *n*
	Zero Order	First Order	Higuchi	Korsmeyer–Peppas		
**CA16**	0.9545	0.8342	0.9838	0.9936	0.0158	0.6234
**CM12**	0.9236	0.858	0.9954	0.9947	0.0025	0.5907

**Table 5:** PT, INR and fibrinogen values.

Parameter	Blood	CA6A	CA10A	CM6A	CM10A
**PT (s)**	13.1 ± 1.2	10. 2 ± 0.5	10. 2 ± 0.5	11.3 ± 0.4	11.0 ± 0.5
**INR**	1.07 ± 0.12	0.70 ± 0.13	0.70 ± 0.16	0.79 ± 0.18	0.76 ± 0.21
**Fibrinogen (mg/dl)**	390 ± 15	460 ± 21	421 ± 12	447 ± 21	410 ± 33
